# IL-4-Primed Microglial Extracellular Vesicles Attenuate Rotenone-Induced Cell Death in SH-SY5Y Cells: A Contributory Role for miR-191-5p

**DOI:** 10.1080/17590914.2026.2712448

**Published:** 2026-08-07

**Authors:** Kemal Ugur Tufekci, Burak I. Arioz, Aysen Cotuk, Cagla Kiser, Bilgesu Ozturk, Tutku Yaras, Gokhan Karakulah, Pembe Keskinoglu, Alper Bagriyanik, Sermin Genc

**Affiliations:** a Izmir Biomedicine and Genome Center, Izmir, Türkiye; b Department of Health Care Services, Vocational School of Health Services, Izmir Democracy University, Izmir, Türkiye; c Center for Brain and Neuroscience Research, Izmir Democracy University, Izmir, Türkiye; d Department of Medical Biology, Faculty of Medicine, Izmir Democracy University, Izmir, Türkiye; e Izmir International Biomedicine and Genome Institute, Dokuz Eylul University, Izmir, Türkiye; f Department of Biostatistics and Medical Informatics, Faculty of Medicine, Dokuz Eylul University, Izmir, Türkiye; g Department of Histology and Embryology, Faculty of Medicine, Dokuz Eylul University, Izmir, Türkiye; h Department of Neuroscience, Health Sciences Institute, Dokuz Eylul University, Izmir, Türkiye

**Keywords:** Extracellular vesicle, hsa-miR-191-5p, IL-4, microglia, rotenone-induced neuronal damage

## Abstract

Microglia contribute to central nervous system homeostasis and neuroprotection partly through the release of small extracellular vesicles (sEVs) carrying regulatory cargoes such as microRNAs. Interleukin-4 (IL-4) alters microglial state and secretory output; however, whether sEVs released from IL-4-treated microglia protect neurons against toxic injury, and which cargoes mediate these effects, remains unclear. Here, we investigated the protective effects of sEVs derived from the IL-4-treated HMC3 human microglial cell line in a rotenone-induced injury model in the SH-SY5Y cell line and examined the contribution of microRNA-191-5p to neuroprotection. Small RNA sequencing revealed a distinct miRNA profile in IL-4-sEVs, with microRNA-191-5p emerging as the most statistically significant upregulated candidate. Its enrichment was confirmed by RT-qPCR. PKH67-labeled sEV-associated fluorescence was detected in SH-SY5Y cells, indicating uptake of microglia-derived sEVs by recipient cells. Functionally, pretreatment with IL-4-sEVs significantly reduced rotenone-induced cell death and preserved cell morphology compared with untreated and control sEV-treated cells. To assess the contribution of microRNA-191-5p, IL-4-sEVs were loaded with a microRNA-191-5p antagomir, which reduced microRNA-191-5p levels and partially attenuated the protective effect of IL-4-sEVs. Together, these findings suggest that sEVs derived from the IL-4-treated HMC3 microglial cell line mitigate rotenone-induced injury in the SH-SY5Y cell line in vitro and that microRNA-191-5p contributes, at least in part, to this effect.

## Background

Microglia, the resident immune cells of the brain, are involved in crucial physiological functions and pathological conditions of the central nervous system (CNS). The primary functions of microglia include immune surveillance, phagocytosis, facilitating neuronal survival, regulating cell death, and promoting synaptogenesis (Borst et al., 2021). Furthermore, microglia can adapt to altering environmental conditions, allowing them to dynamically adapt their state in response to environmental cues (Orihuela et al., 2016). Microglia exposed to IL-4 promote the phagocytosis of cellular and metabolic residues, such as unnecessary cells and misfolded proteins, supporting extracellular matrix reconstruction and promoting neuron survival by inducing neurotrophic factors (Tang & Le, 2016). In addition, they modulate the plasticity of neurons, thereby forming neural circuits (Colonna & Butovsky, 2017).

A wide range of studies have examined the function of sEVs secreted by microglia in the CNS. sEVs are membrane-enclosed vesicles released into the extracellular environment by almost all types of cells (Doyle & Wang, 2019). sEVs have numerous essential functions in the CNS, such as influencing cell growth and survival, supporting neuronal plasticity and tissue homeostasis by triggering the surface receptors of recipient cells, or delivering their cargo content (Schnatz et al., 2021). sEVs include various molecules, such as proteins, lipids, and nucleic acids (Di Bella, 2022), and several nucleic acids, including mRNAs, miRNAs, and other noncoding RNAs, have been found within the contents of sEVs (Hessvik & Llorente, 2018). Furthermore, sEVs can contribute to cell-to-cell communication in the CNS by modifying the metabolic state and physiology of recipient cells through the transport of their cargo molecules (Bang & Thum, 2012). Microglia-derived sEVs play an active role in the pathogenesis of various neurodegenerative diseases, including Alzheimer’s disease (AD), Parkinson’s disease (PD), and amyotrophic lateral sclerosis (ALS), by altering the physiological and metabolic states of recipient cells (Brites & Vaz, 2014). Furthermore, microglial sEVs exert profound effects on the physiological and pathological conditions of neuronal cells by mediating apoptosis, neurogenesis, cell proliferation, and neuroinflammation (Pascual et al., 2020).

Some studies have focused on miRNAs detected in microglial sEVs and their therapeutic effects on CNS diseases (Panaro et al., 2020). Recent research has indicated that microglial sEV-derived miRNAs, such as miR-124, play vital roles in microglia–neuronal crosstalk by affecting neuronal function and cell death (Huang et al., 2018). This study aimed to profile miRNAs enriched in sEVs derived from IL-4-treated HMC3 microglial cells and to evaluate their contribution to neuroprotection in a rotenone-induced injury model in SH-SY5Y cells. We identified several differentially expressed miRNAs in IL-4-sEVs and selected miR-191-5p for further investigation as a candidate mediator of neuroprotection. We then evaluated whether miR-191-5p contributes to the protective effects of IL-4-sEVs in a rotenone-induced injury model in SH-SY5Y cells.

## Methods

### Cell Culture and Treatments

The human microglial HMC3 cell line (RRID:CVCL_II76) was obtained from the American Type Culture Collection (ATCC; Manassas, VA). HMC3 cells were cultured in Dulbecco’s modified Eagle’s medium (DMEM) (Gibco, USA) enriched with 10% fetal bovine serum (FBS), 100 U/ml penicillin, and 100 µg/ml streptomycin (Thermo Scientific, Inc., USA) in a humidified incubator with 5% CO_2_ at 37 °C. The complete DMEM was replenished twice a week, and the cells were passaged once they reached 80–90% confluency. To generate an IL-4-treated microglial condition, HMC3 cells were maintained in medium containing exosome-depleted FBS and then exposed to 10 ng/ml IL-4 in serum-free medium for 24 h (Makita et al., 2015).

### Microglia-Derived Extracellular Vesicle Isolation

At the end of IL-4 treatment, the conditioned media were collected for sEV isolation. The conditioned media were sequentially centrifuged at 3,500 × g for 10 min, twice at 4,500 × g for 10 min each, and then at 10,000 × g for 30 min at 4 °C. The clarified supernatant was transferred to ultracentrifugation tubes and centrifuged at 100,000 × g for 1 h at 4 °C (Chatterjee et al., 2023). The supernatant was discarded, and the pellet was washed with phosphate-buffered saline (PBS), followed by a second ultracentrifugation step at 100,000 × g for 1 h at 4 °C. The final sEV pellet was resuspended in 100 µl PBS, and the sEV suspension was stored at −80 °C.

### Quantitative RT-PCR

To determine the changes in the mRNA levels of selected IL-4-responsive microglial markers (CD206 and HLA-DR), total RNA was isolated from the HMC3 cell line via TRIzol reagent (Invitrogen, USA) according to the manufacturer’s protocol. For cDNA synthesis, the High-Capacity cDNA Reverse Transcription Kit (Applied Biosystems, Thermo, USA) was used according to the manufacturer’s protocol. Quantitative RT–PCR (RT–qPCR) was performed via the GoTaq^®^ qPCR Master Mix Kit on an ABI 7500 Fast Instrument (Applied Biosystems) according to the manufacturer’s instructions. The endogenous glyceraldehyde-3-phosphate dehydrogenase (GAPDH) gene was used to normalize the data. The primer list, including the primer sequences for CD206, HLA-DR, and GAPDH, is presented in Table 1.

**Table 1.** The list of sequences of primers used in quantitative RT‒PCR analysis.

Primer name		Primer sequences (5′–3′)
CD206	Forward	TCT TTG CCT TTC CCA GTC TCC
	Reverse	TGA CAC CCA GCG GAA TTT C
HLA-DR	Forward	AGT CCC TGT GCT AGG ATT TTT CA
	Reverse	ACA TAA ACT CGC CTG ATT GGT C
GAPDH	Forward	ACC ACA GTC CAT GCC ATC AC
	Reverse	TCC ACC CTG TTG CTG TA

For miR-191-5p fold change quantification, the miRNeasy Micro isolation kit (Qiagen, Germany) was used to isolate total RNA from sEVs according to the manufacturer’s instructions. cDNA synthesis was performed via the miRCURY RT Kit (Qiagen, Germany) according to the manufacturer’s protocol. A miRCURY SYBR Green PCR Kit (Qiagen, Germany) was used for RT–qPCR on an ABI7500 Fast Instrument (Applied Biosystems) according to the manufacturer’s protocol. For sEV miRNA quantification, expression was normalized to spike-in cel-miR-39. Relative expression levels of both mRNAs and miRNAs were determined according to the comparative threshold cycle (2^−ΔΔCt^) method.

### Western Blot Analysis

sEV characterization was performed in accordance with the MISEV2023 guidelines (Welsh et al., 2024), as described below. Briefly, the sEV suspension was lysed in RIPA buffer. The protein concentration in the sEVs was determined via a BCA protein assay kit (Takara, Japan) according to the manufacturer’s instructions. sEV proteins were separated via 10% SDS–PAGE and transferred to a polyvinylidene fluoride (PVDF) membrane (Sigma–Aldrich, USA). The membrane was blocked with 1–3% milk powder (Bioshop, Canada) suspended in Tris-phosphate-buffered saline with 0.05% Tween-20 (TBS-T) for 45 min at room temperature. Then, the membranes were incubated with primary antibodies against CD9 (Proteintech, 20597-1-AP, RRID:AB_2878706), CD81 (Santa Cruz, sc-9158, RRID:AB_638255), CD63 (Santa Cruz, sc-5275, RRID:AB_627877) and calnexin (Santa Cruz, sc-23954, RRID:AB_626783) overnight at 4 °C. The next day, the membranes were incubated with horseradish peroxidase (HRP)-conjugated goat anti-rabbit IgG (Cell Signaling Technology, 7074, RRID:AB_2099233) or horse anti-mouse IgG (Cell Signaling Technology, 7076, RRID:AB_330924) secondary antibodies at a dilution of 1:2000 at room temperature for 60 minutes. Finally, the membranes were visualized via the HRP substrate (Millipore, USA).

### Nanoparticle Tracking Analysis

The size distribution of sEVs derived from control and IL-4-treated HMC3 cells was analyzed by nanoparticle tracking analysis using a NanoSight NS300 system (Malvern Panalytical, United Kingdom) at HUNITEK, Hacettepe University, Türkiye. The sEV sample was suspended in prefiltered PBS, and dilutions were performed at ratios of 1:10, 1:100, and 1:10,000 to achieve sEV concentrations between 2 × 10^8^ and 2 × 10^9^ per ml (Ontoria-Oviedo et al., 2018).

### Scanning Transmission Electron Microscopy

To determine the morphology of HMC3 microglia-derived sEVs, isolated sEVs were diluted in PBS, loaded onto a copper mesh, and fixed with 4% paraformaldehyde (Ontoria-Oviedo et al., 2018). The sample was contrasted with 2% uranyl acetate. The sEVs were imaged in STEM mode on a Zeiss Sigma 500 FESEM (STEM-in-SEM), which is distinct from conventional TEM.

### Next-Generation Sequencing Analysis

Small RNA sequencing library preparation and sequencing were performed by Norgen Biotek (Canada). Raw sequencing data were subsequently processed and analyzed in-house. Raw reads were obtained after FASTQC quality control via the tool (https://www.bioinformatics.babraham.ac.uk/projects/fastqc/, RRID:SCR_014583). The 3′ adapter sequences in the raw reads were subsequently removed via the Cutadapt (v2.4, RRID:SCR_011841) command “cutadapt -a TGGAATTCTCGGGTGCCAAGG -o”. Adapter-trimmed reads were mapped to the human GRCh38 reference genome using the mapper.pl module of miRDeep2 (RRID:SCR_010829) (Li et al., 2021). In this process, miRNA reads with fewer than 18 nucleotides were excluded from the analysis. Hairpin and mature miRNA sequences were downloaded via the MirBase (v22, RRID:SCR_003152) database, and the expression levels of known miRNAs for each sample were detected within these sequences. The miRNA expression levels were determined via the miRDeep2 module of the software miRDeep2.pl (Friedländer et al., 2012). The “limma” package (RRID:SCR_010943) was subsequently used in the R (v3.5.1, RRID:SCR_001905) statistical programming language to calculate the expression alterations via the limma-voom function. miRNAs with logFC > 0.6 or < −0.6 and FDR < 0.05 were considered differentially expressed.

### KEGG Pathway Enrichment and Gene Ontology Analysis

The experimentally validated target genes of hsa-miR-191-5p were retrieved from the TarBase (RRID:SCR_010841) and miRTarBase (RRID:SCR_017355) databases using the multiMiR R package (v1.34.0, RRID:SCR_028723), restricting the analysis to validated miRNA–target interactions (Ru et al., 2014). These genes were mapped to Entrez and Ensembl identifiers, and Gene Ontology (GO) and KEGG pathway enrichment analyses were performed using clusterProfiler (v4.20.0, RRID:SCR_016884), with the org.Hs.eg.db (v3.23.1, RRID:SCR_024739), AnnotationDbi (v1.74.0, RRID:SCR_023487), GO.db (v3.23.1, RRID:SCR_024241), and KEGGREST (v1.52.0, RRID:SCR_026949) packages. Terms and pathways with an adjusted *p* value < 0.05 were considered significantly enriched. The GO results were visualized as a bar plot and the KEGG results as a dot plot. Separately, a miRNA–mRNA network for hsa-miR-191-5p was constructed using miRWalk (RRID:SCR_016509) (Sticht et al., 2018), which integrates the TargetScan (RRID:SCR_010845), miRDB (RRID:SCR_010848), and miRTarBase databases, and visualized with Cytoscape (RRID:SCR_003032) (Shannon et al., 2003).

### SH-SY5Y Cell Culture

The human neuroblastoma SH-SY5Y cell line (RRID:CVCL_0019) was cultured with DMEM-Nutrient Mixture F-12 (DMEM/F-12, Gibco, USA) enriched with 10% FBS (Sigma Aldrich, USA), 100 U/ml penicillin, and 100 µg/ml streptomycin (Thermo Scientific, Inc., USA) in an incubator with 5% CO_2_ at 37 °C. For the experimental conditions, SH-SY5Y cells (20,000 cells/well) were seeded in 96-well plates coated with 4.8 µg/ml laminin (Sigma Aldrich, USA). Before sEV treatment, the cell medium was changed to EV-free medium. The cells were incubated first with 20 µg/ml sEVs for 24 hours and then with rotenone (100 µM) for 24 hours to induce neuronal damage (Mursaleen et al., 2023).

### Labeling and Cellular Uptake of sEVs

Isolated sEVs were fluorescently labeled via the PKH67 Green Fluorescent Cell Linker Kit for General Cell Membrane Labeling (Sigma Aldrich, USA) according to the manufacturer’s protocol. Briefly, the sEVs were stained with PKH67 dye, and SH-SY5Y cells were incubated with PKH67-labeled sEVs for 24 hours before visualization via fluorescence microscopy (Olympus IX71, Japan). The data are presented as the ratio of PKH67-positive cells to total cells for quantification.

### sEV Transfection with the hsa-miR-191-5p Inhibitor

The hsa-miR-191-5p inhibitor (antagomir) or negative-control inhibitor (IDT, Integrated DNA Technologies, USA; 100 µM stock in TE buffer) was loaded into isolated sEVs using Lipofectamine RNAiMAX (Invitrogen, USA), adapted from Shtam et al. (Shtam et al., 2013). Briefly, 2 µl of inhibitor (or negative control), 6 µl of Lipofectamine RNAiMAX, and 92 µl of siRNA buffer were combined and incubated for 10 min at room temperature to allow complex formation. Then, 300 µl of the sEV suspension was added to a final volume of 400 µl, giving a final sEV concentration of approximately 100 µg/ml and a final inhibitor concentration of 500 nM, and the mixture was incubated for 30 min at room temperature. After incubation, the samples were filtered through a 0.22 µm filter and subsequently held at 4 °C for 30–60 min to reduce residual transfection-reagent toxicity. Transfection efficiency was assessed by RT-qPCR.

### Propidium Iodide Staining

Propidium iodide (PI) (Sigma–Aldrich, USA) was used to stain the DNA structure of dead cells with damaged membranes (Kabakov & Gabai, 2018). At the end of the treatments, PI was added to each sample well and incubated for approximately 15 minutes in the dark in an incubator. Then, PI-stained (PI-positive) cells were visualized via a fluorescence microscope (Olympus IX-71, Japan), and quantification was performed via ImageJ software (RRID:SCR_003070). Finally, the data are expressed as the ratio of the number of PI-positive cells to the total number of cells.

### Statistical Analysis

For all the statistical analyses, GraphPad Prism 8.0 (GraphPad Software, Inc., CA, USA, RRID:SCR_002798) was used. To test nonparametric and parametric data, the Mann–Whitney *U* test and Student’s *t* test, respectively, were used. One-way ANOVA with the Bonferroni multiple comparison correction was used to compare multiple groups with a normal distribution. Furthermore, the Kruskal–Wallis test with Dunn’s multiple comparison test was used to compare multiple groups without a normal distribution. All the data are presented as the means ± standard errors of the means. In all the experiments, a *p* value of <0.05 was considered to indicate statistical significance. All experiments were performed in at least three independent biological replicates unless otherwise stated. Each biological replicate was derived from a separate cell culture preparation and/or independent sEV isolation. Technical replicates, when present, were averaged before statistical analysis.

## Results

### IL-4 Induction in HMC3 Microglia and Characterization of HMC3-Derived sEVs

**Figure 1.**
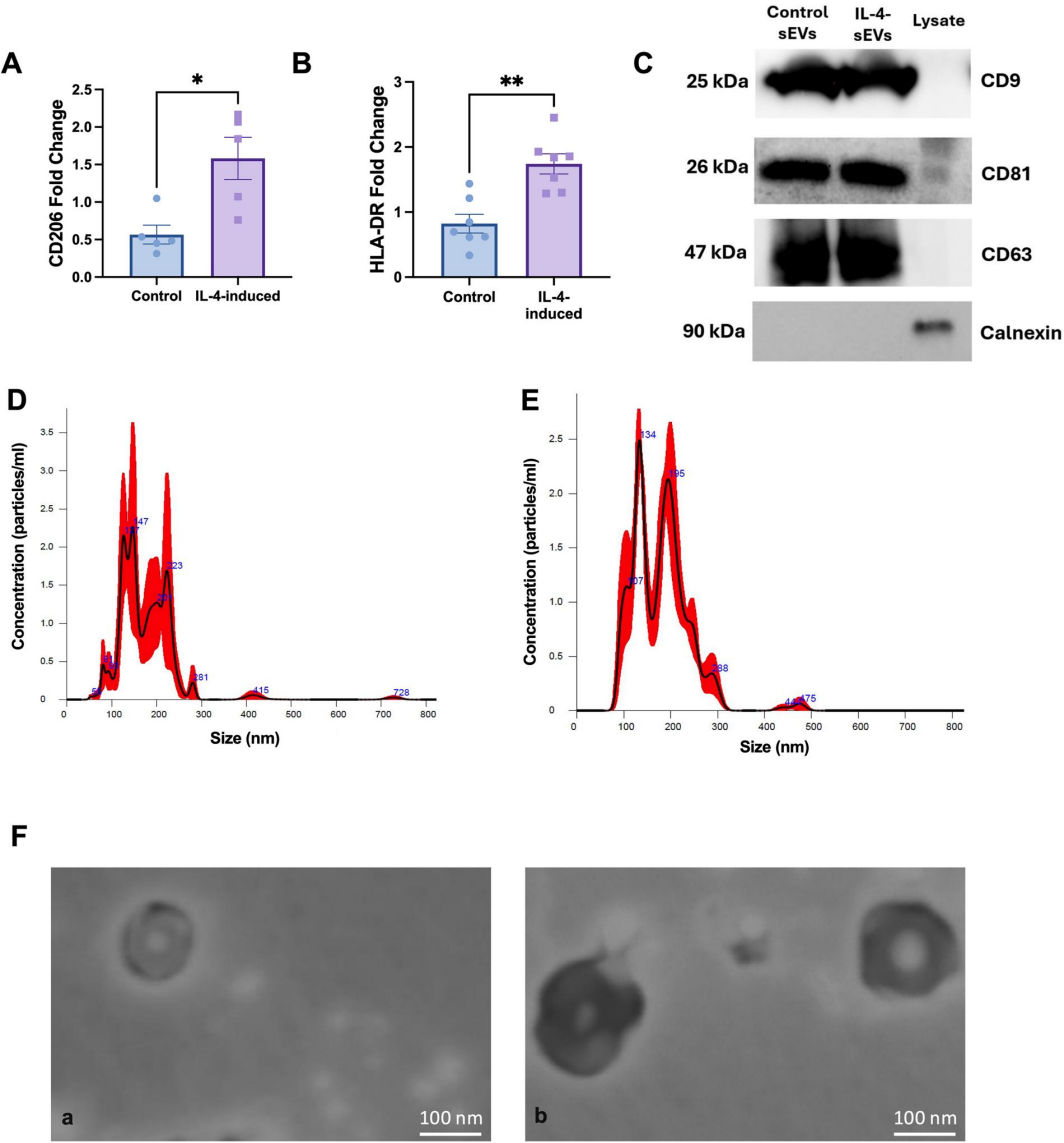
Characterization of sEVs derived from IL-4-treated HMC3 cells. mRNA expression of (a) CD206 and (b) HLA-DR in HMC3 cells upon 10 ng/ml IL-4 treatment. (c) Western blot analysis of the presence of EV markers and the absence of non-EV markers. The size distributions of (d) sEVs derived from control HMC3 cells and (e) sEVs derived from IL-4-treated HMC3 cells were determined by nanoparticle tracking analysis (NTA). (f) Representative STEM-in-SEM images of isolated sEVs showing individual cup-shaped, membrane-enclosed vesicles (panels a and b). Scale bars, 100 nm.

HMC3 cells were treated with 10 ng/ml IL-4 for 24 h, and the expression of selected IL-4-responsive markers was evaluated by RT-qPCR. IL-4 treatment increased the expression of CD206, a canonical IL-4-responsive marker, and also increased HLA-DR (Figure 1A, B). These changes indicate an IL-4-responsive shift in HMC3 marker expression, primarily reflected by increased CD206.

Western blot, STEM imaging and nanoparticle tracking analysis (NTA) were used to characterize the sEVs. The presence of CD9, CD81, and CD63 and the absence of calnexin were used as markers to characterize sEVs. CD9, CD81, and CD63 were detected in sEVs derived from both control and IL-4-treated HMC3 cells. In contrast, calnexin was not detected in sEVs derived from either control or IL-4-treated HMC3 cells, consistent with the absence of cellular contamination. The corresponding HMC3 cell lysate was used as a non-sEV control, in which calnexin was detected, whereas EV markers were not enriched (Figure 1C). The size range of sEVs derived from control and IL-4-treated HMC3 cells was 100–250 nm in diameter, with a multimodal distribution, according to NTA (Figure 1D, E), consistent with the polydispersity expected of sEV preparations. Furthermore, STEM imaging revealed the characteristic cup-shaped, membrane-enclosed morphology of individual sEVs, with multiple vesicles within the expected size range observed (Figure 1F, panels a and b).

### The miR-191-5p Expression Level in the sEVs of HMC3 Cells Increased after IL-4 Induction

**Figure 2.**
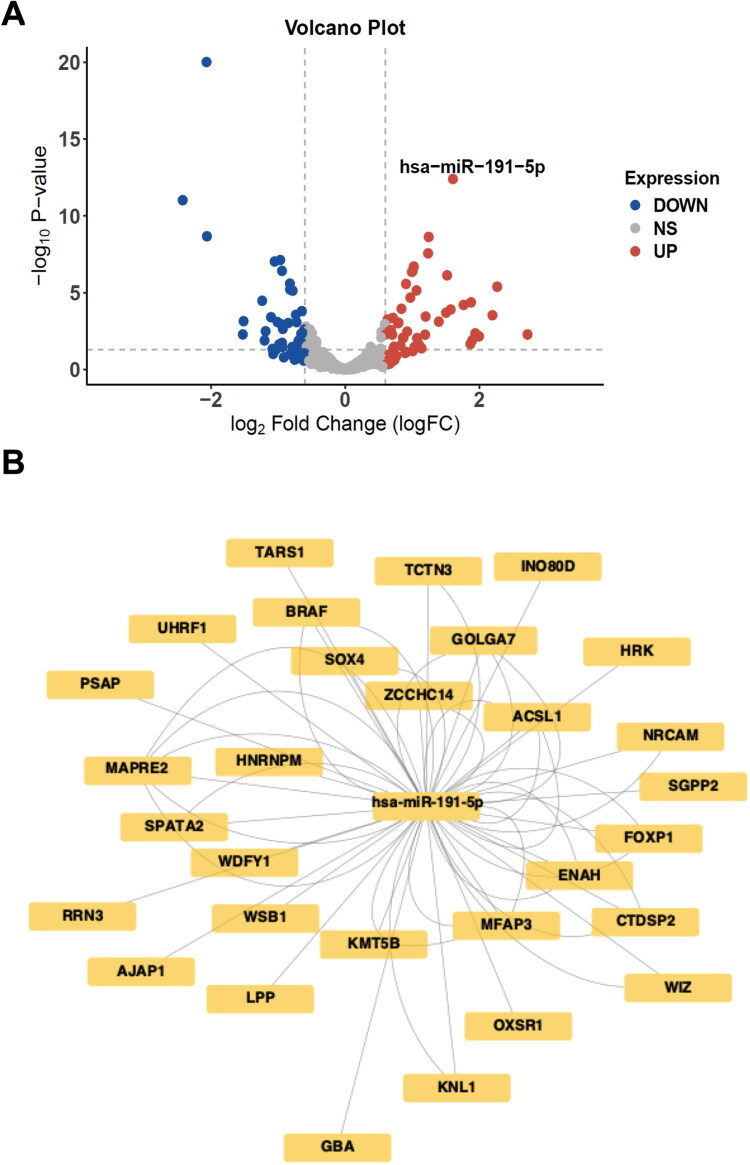
Differentially expressed miRNAs and predicted targets of hsa-miR-191-5p. (a) Volcano plot of differentially expressed miRNAs in control sEVs and IL-4-sEVs. The horizontal axis represents the log2 of the fold change, and the vertical axis represents the -log10 of the *p* value, with hsa-miR-191-5p indicated. Blue, downregulated; red, upregulated; grey, not significant (NS). (b) miRNA–mRNA network analysis of hsa-miR-191-5p, showing 30 predicted candidate mRNA targets.

We performed miRNA analysis via next-generation sequencing (NGS) to assess changes in miRNA expression levels in control sEVs and IL-4-sEVs. According to the NGS results, a total of 275 miRNAs were detected in both the control sEV and IL-4-sEV groups. Differential expression analysis identified 31 miRNAs that met the predefined cutoff criteria between control sEVs and IL-4-sEVs (Figure 2A). We identified 21 upregulated miRNAs in IL-4-sEVs, of which the top five are listed in Table 2. In addition, 10 miRNAs were downregulated, and the top five are listed in Table 3. hsa-miR-191-5p showed the strongest statistical significance among the upregulated miRNAs in IL-4-sEVs and has previously been linked to neuroprotection; it was therefore selected for follow-up validation. miR-374a-5p, although the most significantly altered miRNA overall (logFC = −2.07, FDR = 3.38 × 10–18), was downregulated in IL-4-sEVs and was not pursued, as this study focused on upregulated cargo. Additionally, miRNA–mRNA network analysis predicted 30 candidate mRNA targets for hsa-miR-191-5p (Figure 2B; targets with at least one hit in any miRWalk database). These predictions were not experimentally validated and are presented as hypothesis-generating.

**Table 2.** The list of upregulated miRNAs in sEVs derived from IL-4-treated HMC3 cells.

miRNA	logFC	FDR
hsa-miR-191-5p --pr-- hsa-mir-191	1.606823	7.00E-11
hsa-miR-423-5p --pr-- hsa-mir-423	1.245283	1.66E-07
hsa-miR-125a-5p --pr-- hsa-mir-125a	1.236558	1.60E-06
hsa-miR-151a-5p --pr-- hsa-mir-151a	1.023619	7.35E-06
hsa-miR-151b --pr-- hsa-mir-151a	1.02264	7.35E-06

logFC: log(fold change), FDR: false discovery rate, sorted according to FDR.

**Table 3.** List of downregulated miRNAs in sEVs derived from IL-4-treated HMC3 cells.

miRNA	logFC	FDR
hsa-miR-374a-5p --pr-- hsa-mir-374a	−2.06713	3.38E-18
hsa-miR-27a-5p --pr-- hsa-mir-27a	−2.42244	1.11E-09
hsa-miR-320a-5p --pr-- hsa-mir-320a	−2.06128	1.66E-07
hsa-miR-577 --pr-- hsa-mir-577	−1.05165	4.09E-06
hsa-miR-619-5p --pr-- hsa-mir-619	−1.23708	0.000536

logFC: log(fold change), FDR: false discovery rate, sorted according to FDR.

**Figure 3.**
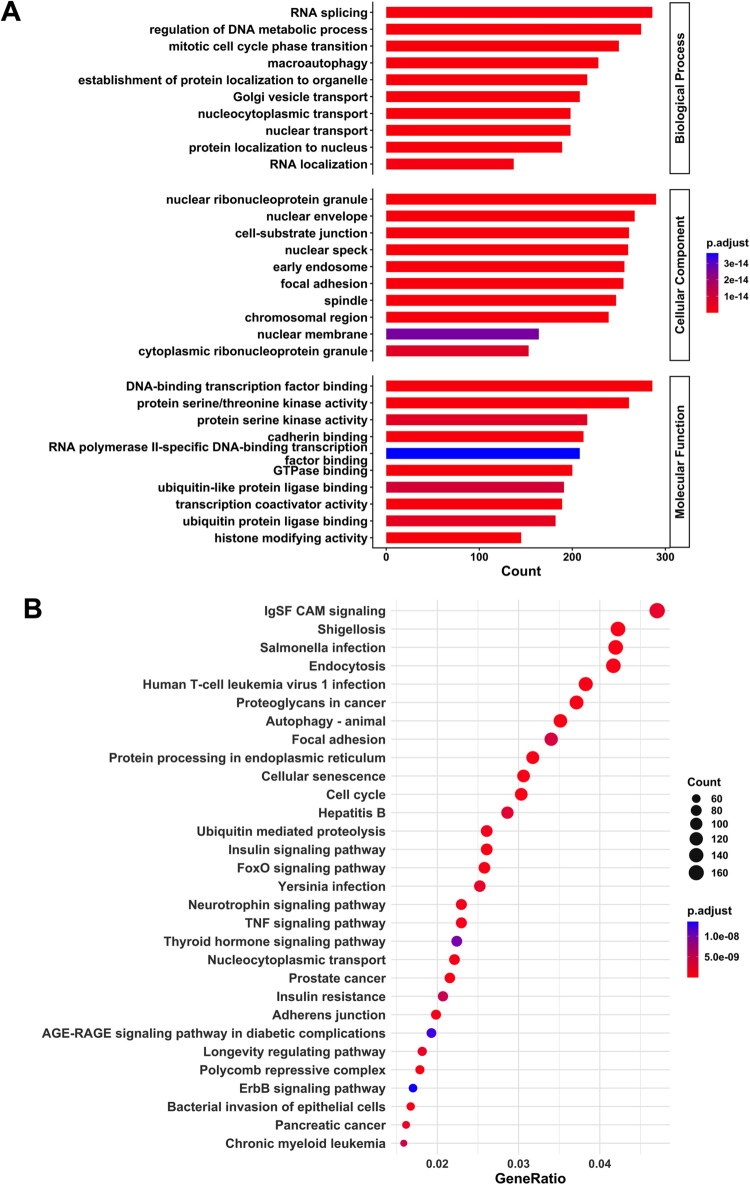
Functional enrichment analysis of miRNA target genes. (a) Gene Ontology (GO) enrichment analyses of validated miRNA targets are displayed in three categories: biological process, cellular component, and molecular function showing the top enriched terms. (b) KEGG pathway enrichment analysis of validated miRNA targets, showing the top 30 enriched pathways.

The validated target genes of the differentially expressed miRNAs were subjected to GO enrichment and KEGG pathway analyses. GO enrichment analysis was performed across the three ontologies (Figure 3A). The most significantly enriched terms included nucleocytoplasmic and nuclear transport, macroautophagy, and RNA splicing (biological process); nuclear speck, cell–substrate junction, focal adhesion, and early endosome (cellular component); and DNA-binding transcription factor binding, cadherin binding, and protein serine/threonine kinase activity (molecular function). KEGG pathway analysis identified 168 significantly enriched pathways (all *p* adjusted < 0.05). The most enriched terms (Figure 3B) were dominated by core cellular processes, with autophagy as the single most enriched pathway, alongside FoxO, neurotrophin, and TNF signaling, ubiquitin-mediated proteolysis, endocytosis, longevity regulation, and apoptosis. Although they ranked outside the top 30 by gene ratio and are therefore not displayed in Figure 3B, neurodegeneration-related pathways were also significantly enriched, including pathways of neurodegeneration—multiple diseases (*p* adjusted = 1.6 × 10^−8^), Alzheimer disease (3.6 × 10^−8^), amyotrophic lateral sclerosis (7.0 × 10^−8^), Huntington disease (1.2 × 10^−5^), prion disease (5.3 × 10^−6^), Parkinson disease (1.1 × 10^−4^), and the dopaminergic synapse (8.0 × 10^−4^).

### PKH67-Labeled HMC3-Derived sEVs Were Detected in SH-SY5Y Cells

**Figure 4.**
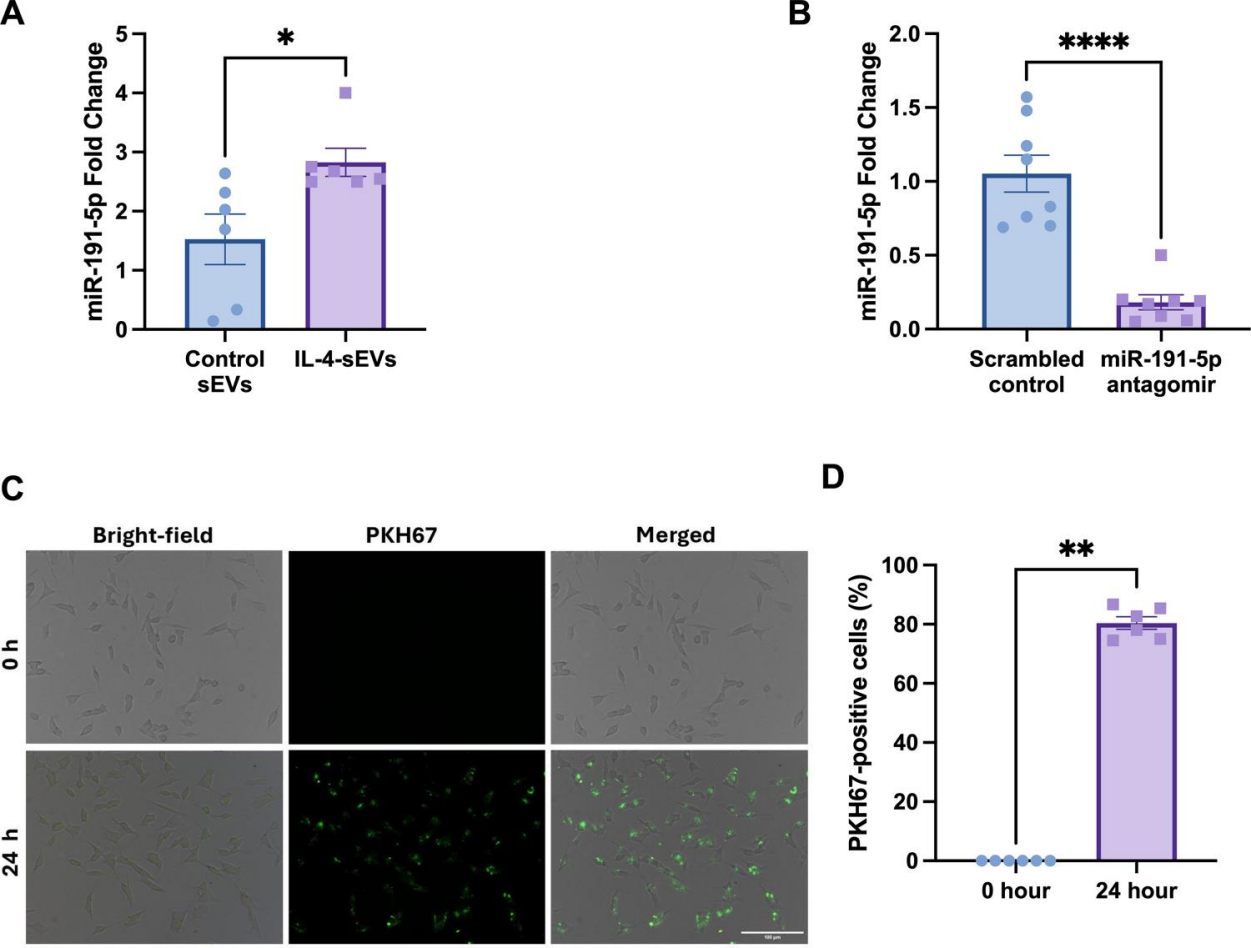
miR-191-5p validation and PKH67-labeled sEV uptake assay. (a) The level of miR-191-5p in IL-4-sEVs. (b) The level of miR-191-5p in IL-4-sEVs transfected with the negative control or the miR-191-5p antagomir. (c) Fluorescence imaging and (d) quantification of PKH67-positive cells (%) revealed that PKH67-positive fluorescence was detected in SH-SY5Y cells after incubation with labeled sEVs. The data are presented as the means ± SEM (n = 6 for a and d; n = 8 for b). Pairwise comparisons were performed by the Mann–Whitney U test; *p < 0.05, **p < 0.01, ****p < 0.0001.

An increase in miR-191-5p expression in sEVs upon IL-4 treatment was validated by RT–qPCR (Figure 4A). SH-SY5Y cells were incubated with PKH67-labeled HMC3-derived sEVs for 24 h, and PKH67-positive intracellular fluorescence was detected at the 24 h endpoint (Figure 4C). Approximately 80% of SH-SY5Y cells were PKH67-positive under these conditions (Figure 4D).

### IL-4-sEVs Attenuated Rotenone-Induced Neuronal Cell Death

**Figure 5.**
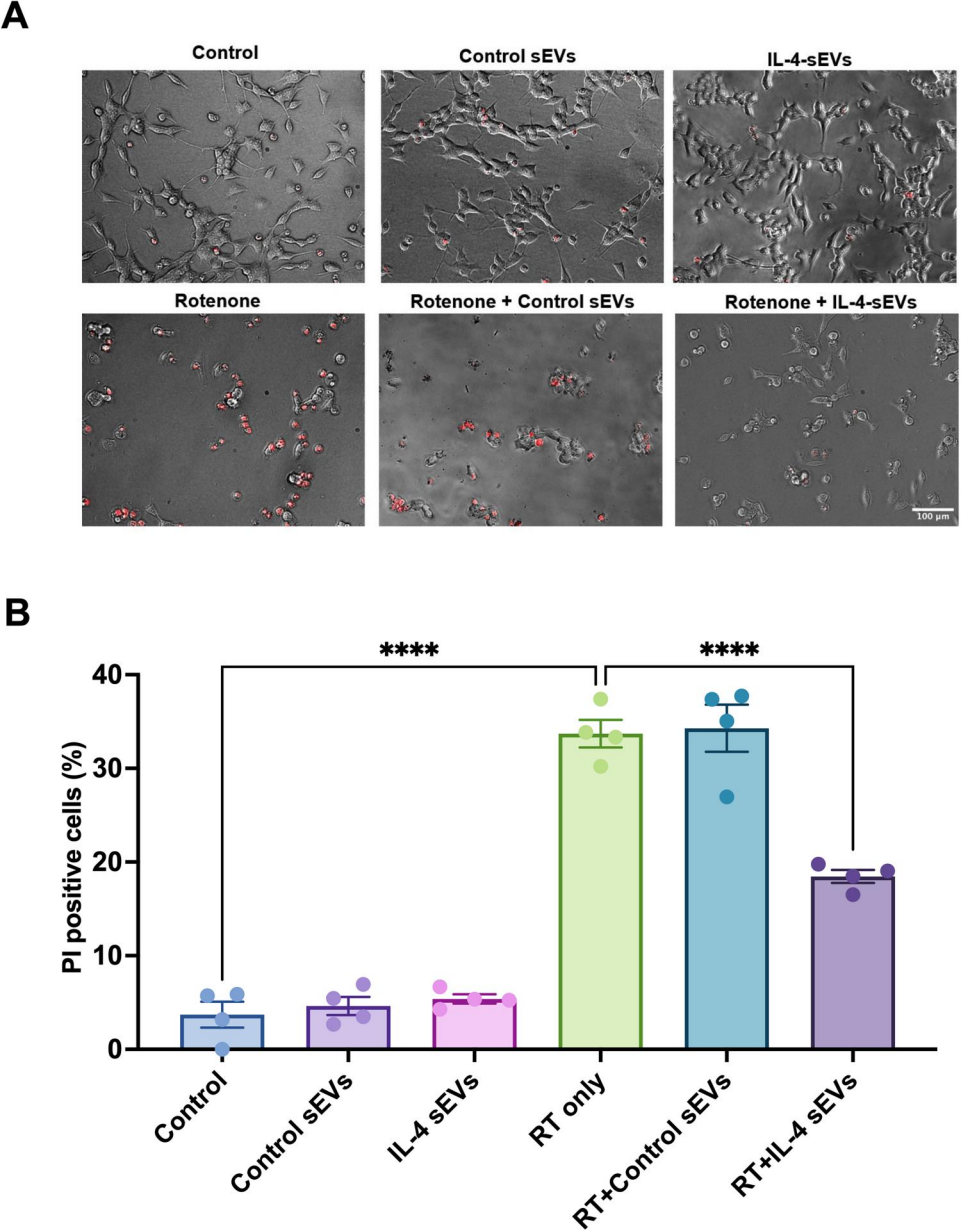
IL-4-sEVs reduce neuronal cell death during rotenone-induced neuronal damage. (a, b) IL-4-sEVs reduced the percentage of PI-positive rotenone-treated SH-SY5Y cells. The data are presented as the means ± SEM (n = 4, independent biological replicates from four independent experiments). Group comparisons were performed by one-way ANOVA with Bonferroni correction; ****p < 0.0001.

To investigate whether control sEVs or IL-4-sEVs affect neuronal cell death, SH-SY5Y cells were stained with PI to assess cell death in response to sEVs and rotenone treatment. The results revealed that rotenone (100 µM) treatment significantly induced cell death, by 33.69%, in SH-SY5Y cells, and cell death was considerably suppressed by IL-4-sEV pretreatment compared with that in the Rotenone group (Figure 5A, B). These results indicate that pretreatment with IL-4-sEVs reduced rotenone-induced cell death in SH-SY5Y cells.

### hsa-miR-191-5p is Associated with the Neuroprotective Effect of IL-4-sEVs on Cell Death

**Figure 6.**
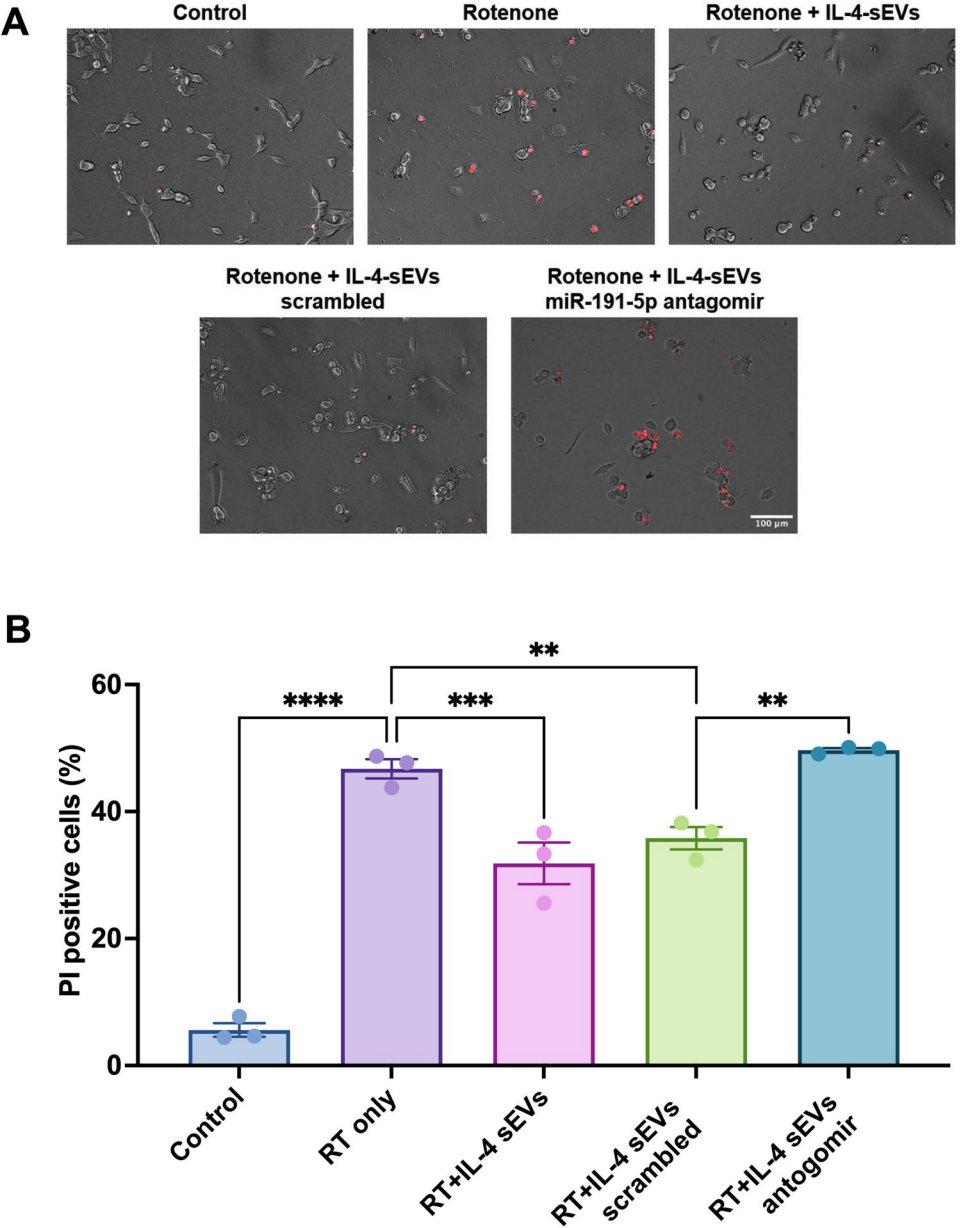
Inhibition of miR-191-5p partially attenuated the protective effect of IL-4-sEVs on neuronal cell death. (a, b) Inhibition of miR-191-5p in IL-4-sEVs partially attenuated the protective effect of IL-4-sEVs on neuronal cell death. The data are presented as the means ± SEM (n = 3, independent biological replicates from three independent experiments). Group comparisons were performed by one-way ANOVA with Bonferroni correction; **p < 0.01, ***p < 0.001, ****p < 0.0001.

SH-SY5Y cells were stained with PI to determine whether inhibition of miR-191-5p in IL-4-sEVs alters their protective effect against rotenone-induced neuronal cell death. The results revealed that rotenone (100 µM)-induced neuronal damage increased the degree of death of SH-SY5Y cells by up to 45.20% (Figure 6A, B). We transfected sEVs with a negative control and miR-191-5p antagomirs. The miR-191-5p expression level in IL-4-sEVs was significantly decreased upon transfection with the miR-191-5p antagomir (Figure 4 B). IL-4-sEV treatment significantly reduced cell death, whereas miR-191-5p inhibition partially reversed the decrease in cell death (Figure 6A, B). These results suggest that miR-191-5p contributes, at least in part, to the protective effect of IL-4-sEVs against rotenone-induced cell death in SH-SY5Y cells.

## Discussion

In this study, we demonstrated that the levels of several miRNAs were altered in sEVs derived from IL-4-treated HMC3 microglial cells compared with those derived from untreated control cells. Among these miRNAs, hsa-miR-191-5p emerged as the most statistically significant upregulated candidate and was selected for further analysis. Our findings support a neuroprotective contribution of hsa-miR-191-5p enriched in IL-4-sEVs in rotenone-treated SH-SY5Y cells.

Microglia, the innate immune cells of the CNS, are pivotal in neuronal development, neurogenesis, axonal remodeling, and synaptic pruning (Hu & Tao, 2024). Under pathological conditions, including neurodegenerative diseases, microglia adopt context-dependent activation states associated with altered cytokine release, phagocytic activity, and trophic support (Nakaso, 2024). IL-4 exposure is known to shift microglial gene expression and secretory output toward a state associated with debris clearance and neuronal support (Guo et al., 2022). We observed increased HLA-DR alongside CD206 in IL-4-treated HMC3 cells. This is somewhat unexpected, as IL-4 typically downregulates MHC class II in primary macrophages and microglia; the immortalized nature of HMC3 may account for this atypical response. We therefore interpret CD206 as the principal IL-4-responsive marker in this system, with the HLA-DR finding warranting cautious interpretation. An increasing number of studies have shown that IL-4-treated microglia exert neuroprotective effects in many brain diseases, such as AD (Wang et al., 2021), PD (Jin et al., 2019), cerebral ischemia (Xue et al., 2021), and intracerebral hemorrhage (Tschoe et al., 2020). Therefore, understanding the neuroprotective effects of IL-4-treated microglia and their underlying mechanisms may help identify new therapeutic targets for neurological diseases and injuries.

Cell-to-cell communication is vital for the maintenance of cellular functions and homeostasis in the CNS. sEVs contribute to microglia and neuron interactions by transporting important molecules, including miRNAs, other nucleic acids, and proteins (Filannino et al., 2024). miRNAs in sEVs may contribute to the neuroprotective effect of IL-4 on microglia. A previous microarray study in IL-4-stimulated primary murine microglia reported broad changes in miRNA expression, including altered levels of miR-145, miR-214, miR-26b, miR-124, and miR-653, among others (Freilich et al., 2013). In our study, small RNA sequencing of sEVs derived from IL-4-treated HMC3 cells revealed that several miRNAs were differentially expressed compared with sEVs derived from control HMC3 cells. Our findings regarding the upregulation of miR-151 and miR-653 were also confirmed by other studies (Freilich et al., 2013; Tian et al., 2019). The upregulated hsa-miR-191-5p was selected for further investigations.

Several IL-4-sEV miRNAs, including miR-124, miR-26, miR-23a-5p, miR-137, miR-135a-5p, and miR-672-5p have shown neuroprotective and reparative effects in models of stroke, cerebral ischemia–reperfusion, and spinal cord injury, supporting the broader concept that IL-4 priming enriches protective sEV cargo. In the present rotenone model, the neuroprotection associated with miR-191-5p may relate to its reported suppression of the NLRP3 inflammasome and regulation of DAPK1, both implicated in mitochondrial-stress-driven neuronal death, although this remains to be tested directly in our system. In our study, we found that the enrichment of hsa-miR-191-5p in IL-4-sEVs was associated with reduced neuronal cell death, and inhibition of miR-191-5p partially attenuated this protective effect. The baseline level of rotenone-induced cell death differed between our two independent experiments (33.69% vs 45.20% PI-positive cells), despite an identical rotenone concentration. This reflects the inter-experimental variability inherent to independent sEV preparations and cell cohorts and underscores the importance of the internal within-experiment controls included in each assay.

hsa-miR-191-5p is a 23-nucleotide-long intragenic miRNA located on chromosome 3 (Zhang et al., 2018). The expression of hsa-miR-191-5p has been shown to be altered in numerous studies related to neurodegenerative and neurological diseases. For example, pioneering research revealed that hsa-miR-191-5p was downregulated in AD patients compared with healthy individuals (Tan et al., 2014). hsa-miR-191-5p has also been reported to be significantly dysregulated in patients with multiple sclerosis (Vistbakka et al., 2018; 2022), autism (Wang, Lu, et al., 2022), attention-deficit/hyperactivity disorder (Sánchez-Mora et al., 2019), fragile X syndrome (Sotoudeh Anvari et al., 2022) and neuromuscular disorders (Mousa et al., 2023). In a sequencing study, hsa-miR-191-5p was reported to be significantly upregulated after ischemic stroke in nonhuman primates (Chen et al., 2021). The role of miR-191-5p in the regulation of the NLRP3 inflammasome has been illustrated (Chen et al., 2021). The overexpression of miR-191-5p inhibits the transcription of NLRP3 and protects primary mouse retinal pigment epithelium cells from amyloid-beta (Aβ)1-40–mediated cell damage. hsa-miR-191-5p was investigated in microglia; Wan et al. reported that hsa-miR-191-5p attenuated microglial injury in primary microglia and hippocampal tissue in a mouse model of AD (Wan et al., 2021). In addition, they reported that the overexpression of hsa-miR-191-5p increased cell viability and decreased the rate of apoptosis caused by Aβ1–42 treatment in microglia. An *in vitro* model of AD indicated that miR-191-5p reduces tau phosphorylation and Aβ generation through the regulation of death-associated protein kinase 1 (DAPK1) (Wang, Shui, et al., 2022). The addition of miR-191-5p induces neurite growth in SH-SY5Y cells. In brain tissue samples from AD patients, low levels of miR-191-5p and upregulated expression of its target gene DAPK1 have been demonstrated (Wang, Shui, et al., 2022). Elevated expression levels of hsa-miR-191-5p were detected in the platelets of patients with isolated REM sleep behavior disorder who developed PD within eight years, suggesting that hsa-miR-191-5p could serve as a biomarker for these patients (Arnaldo et al., 2025). In our study, compared with the scrambled-control-loaded sEVs, hsa-miR-191-5p knockdown increased cell death in SH-SY5Y cells. Taken together, HMC3-derived IL-4-sEVs attenuated rotenone-induced cell death in SH-SY5Y cells, an effect partially mediated by hsa-miR-191-5p.

According to our bioinformatics analyses, hsa-miR-191-5p is associated with neural targets and pathways. Target analysis revealed that neuronal cell adhesion molecule (NRCAM) is involved in several neuronal development events, including the formation of synapses, proliferation, differentiation, axon growth, and guidance (Sakurai, 2012), as well as neuronal death. Neuronal death protein DP5 (HRK) was found to participate in apoptosis signaling in different neuron types (Coultas et al., 2007). Wang et al. demonstrated that the lncRNA ZFAS1 regulates hippocampal cell damage in epilepsy by upregulating the miR-15a-5p/OXSR1/NF-κB axis (Wang, Na, et al., 2022). Furthermore, Potzner et al. demonstrated that Sox4 and Sox11 are fundamentally necessary for the development of the sympathetic nervous system; their deletion led to hypoplastic sympathetic ganglia (Potzner et al., 2010). In the GO enrichment analysis, although the predominant categories corresponded to nuclear transport, RNA processing, and proteostasis-related processes, several neuron-related biological processes were also significantly enriched, including cell morphogenesis involved in neuron differentiation (*p* adjusted = 4.0 × 10–15), regulation of neuron projection development (1.3 × 10^−1^³), axonogenesis (1.9 × 10–11), and axon development (2.2 × 10^−10^), consistent with a role for the miR-191-5p target genes in neuronal structural and developmental programs. KEGG analysis indicated that the target genes converged on core processes governing neuronal proteostasis and stress responses. The most strongly enriched pathway was autophagy, together with ubiquitin-mediated proteolysis, FoxO signaling, longevity regulation, and apoptosis, which are implicated in rotenone-induced mitochondrial stress and dopaminergic neuronal death. Consistent with this, neurodegeneration-related pathways were also significantly enriched, including pathways of neurodegeneration—multiple diseases, Alzheimer disease, amyotrophic lateral sclerosis, Huntington disease, Parkinson disease, and the dopaminergic synapse, albeit with more modest fold enrichment and outside the top 30 by gene ratio, reflecting their substantial gene overlap with these underlying proteostasis and cell-death pathways. Neurotrophin and TNF signaling further implicate neuronal survival and neuroinflammatory axes. Together with the predicted neuronal targets noted above, these associations are consistent with a neuroprotective role for IL-4-sEV–derived miR-191-5p, although they represent bioinformatic predictions requiring experimental validation.

Several limitations should be acknowledged. First, we did not experimentally validate any direct downstream target (e.g., DAPK1, NLRP3, or NRCAM) in rotenone-treated SH-SY5Y cells; the predicted targets identified by our bioinformatic analyses are therefore hypothesis-generating, and confirmation by RT-qPCR, Western blot, and luciferase reporter assays is required to establish the downstream mechanism. Second, the antagomir-loading approach does not fully exclude free-inhibitor carryover or transfection-reagent effects, as Lipofectamine-only-loaded sEV controls, quantification of free-inhibitor carryover, and post-loading re-characterization (NTA, Western blot, electron microscopy) were not performed; these results should therefore be interpreted as supportive rather than definitive. Third, a gain-of-function experiment using a miR-191-5p mimic loaded into control sEVs (required to demonstrate sufficiency), and no-rotenone baselines for the loaded-sEV conditions were not included. Fourth, the model relies on the immortalized HMC3 microglial line and undifferentiated, proliferative SH-SY5Y cells, which lack mature post-mitotic neuronal features and may respond to rotenone differently from mature neurons; validation in differentiated SH-SY5Y, primary, or iPSC-derived neurons and microglia, or *in vivo*, will be needed to strengthen translational relevance. In particular, confirmation that IL-4 stimulation increases miR-191-5p in primary human microglia, and that the neuroprotective effect is reproduced in primary neurons, will be an essential next step; these experiments were beyond the scope of the present in vitro study and are a priority for follow-up work. Fifth, we focused on a single candidate, hsa-miR-191-5p. Several other miRNAs were significantly enriched in IL-4-sEVs, including hsa-miR-423-5p, hsa-miR-125a-5p, and hsa-miR-151a-5p, which may also contribute to the observed protection, potentially in a combinatorial manner. Functional characterization of these additional cargoes represents an important translational opportunity that we intend to pursue. Finally, cell death was assessed by PI positivity normalized to phase-contrast cell counts; DAPI co-staining would provide more precise total-cell enumeration in future studies.

## Conclusions

The present findings suggest that sEVs derived from IL-4-treated HMC3 microglial cells alleviate rotenone-induced cell death in SH-SY5Y cells *in vitro* and that hsa-miR-191-5p contributes, at least in part, to this protective effect. These results provide a rationale for further investigation of IL-4-sEV cargo molecules as potential mediators of neuroprotective signaling. Because only miR-191-5p was functionally examined in this study, the contribution of other differentially expressed sEV miRNAs remains to be determined.

## Data Availability

All the data generated during and/or analyzed in the current study are available from the corresponding author upon reasonable request.

## List of Abbreviations

Aβ: amyloid-beta
AD: Alzheimer’s disease
ALS: amyotrophic lateral sclerosis
BBB: blood–brain barrier
CNS: central nervous system
DAPK1: death-associated protein kinase 1
DMEM: Dulbecco’s modified Eagle’s medium
FBS: fetal bovine serum
HRK: neuronal death protein DP5
IL-4: interleukin-4
miRNAs: microRNAs
NTA: nanoparticle tracking analysis
NSC: neural stem cell
NRCAM: neuronal cell adhesion molecule
PD: Parkinson’s disease
sEVs: small extracellular vesicles
